# NGS-based approach for diagnostically unidentified *Mycobacterium saskatchewanense*, a rare non-tuberculous mycobacterium

**DOI:** 10.3389/fcimb.2025.1685898

**Published:** 2025-12-16

**Authors:** Giulia Gatti, Ludovica Ingletto, Giorgio Dirani, Silvia Zannoli, Francesca Taddei, Claudia Colosimo, Laura Dionisi, Anna Marzucco, Maria Sofia Montanari, Agnese Denicolò, Francesco Congestrì, Laura Grumiro, Martina Brandolini, Massimiliano Guerra, Alessandra Mistral De Pascali, Alessandra Scagliarini, Monica Cricca, Vittorio Sambri

**Affiliations:** 1 Department of Medical and Surgical Sciences-DIMEC, Alma Mater Studiorum, University of Bologna, Bologna, Italy; 2 Operative Unit of Microbiology, The Greater Romagna Hub Laboratory, Cesena, Italy

**Keywords:** bioinformatics, diagnosis, marker, nontuberculous, sequence analysis

## Abstract

**Introduction:**

The implementation of advanced technologies and algorithms for diagnosis and genome analysis has made a fundamental contribution to pathogens’ identification and investigation.

**Methods:**

The study of non-tuberculous mycobacteria (NTM) benefited from a next-generation sequencing (NGS) approach, making it possible to describe sequences of rare pathogens. This study identified 20 diagnostically unknown isolates as *Mycobacterium saskatchewanense* ST 691, an environmental NTM. The isolates were sequenced on two different platforms to compare their throughput and to investigate shared and unique single nucleotide polymorphism (SNP) counts, phylogeny based on concatenated 16S, hsp65, and rpoB genes, and core-genome multilocus sequence typing (MLST), in order to broaden the current knowledge of *Mycobacterium saskatchewanense*.

**Results:**

Principal component analysis on the three genes combined with the mutations’ annotation suggests that rpoB may serve as a suitable marker to distinguish *M. saskatchewanense* from other NTM.

**Discussion:**

Our results show that frontier studies performed using NGS can help in overcoming the limits of traditional diagnostic assays and deepen the knowledge on rare and uncommon NTM that are raising clinical concern.

## 1 Introduction

The term non-tuberculous mycobacteria (NTM) refers to a heterogeneous group that includes various species, except for the ones classified into the *Mycobacterium tuberculosis* complex or the *Mycobacterium leprae*. The distribution of NTM is generally ubiquitous (Jiang et al., 2024), and bacteria can be isolated from different environmental reservoirs such as water and water systems, dust, soil, and related matrixes (Bryant et al., 2016; Falkinham, 2021). Given the wide dissemination and the continuous explosion of likely infecting sources, the incidence of NTM is significantly growing worldwide. This trend may jeopardize fragile patients in which NTM blood-stream dissemination leads to organ dysfunction, disorders, and diverse clinical conditions, posing a challenge for the treatment and management of patients (Abad and Razonable, 2016; Nagata et al., 2020). Despite the significant impact on human health, NTM diagnosis still requires well-defined criteria for identification when microbial culture is insufficient (Tomishima et al., 2022). Considering the ease of contracting the infection, an appropriate and accurate diagnostic approach is needed to identify uncommon environmental NTM (Khosravi et al., 2024). Indeed, to achieve a complete diagnosis, clinical, radiographic, and microbiological data must be combined. Up to date, the microbiological *gold standard* for detecting NTM is the bacterial culture, which is not devoid of deficiencies: *in vitro* isolation can be time-consuming, influenced by low bacterial burden, and by a wide and diverse growth temperature spectrum for each different NTM (Wang et al., 2022). This is mainly reported in cases of recently discovered NTM infections when the microbial culture has a low rate of positivity, and the microbial agent cannot be clearly distinguished through microscopy. PCR-based techniques and serological assays could be considered alternative tests, but they require a preliminary knowledge of the target microorganism (Huang et al., 2023). More recently, these limitations have been overcome by introducing genotyping methods relying on pulsed-field gel electrophoresis, commercial DNA probes, polymerase chain reaction amplification, and restriction-enzyme analysis. In the same manner, early sequencing methods were based on gene-specific investigation, such as *16S* rRNA, *rpoB*, *hsp65*, *dnaj*, *soda*, and *16S*-*23S* internal transcribed spacer (*ITS*) (Tenover et al., 1995; Maleki et al., 2017). The step forward in NTM analysis was the application of a multilocus sequence typing (MLST), core genome MLST (cgMLST), and a next-generation sequencing (NGS) approach characterizing a set of concatenated gene sequences to properly identify NTM species (Khosravi et al., 2024). More recently, next-generation whole-genome sequencing (WGS) emerged as the method of choice, as it allows the evaluation of single-nucleotide polymorphisms (SNPs) and global diversity, by defining SNPs associated with drug resistance, phylogenetic diversity, and differentiation of mixed infections (Dohál et al., 2021; Inkster et al., 2021; Lecorche et al., 2021; Davidson et al., 2022). Additionally, WGS data provide a detailed insight into the molecular epidemiology and definition of clusters of infection (Dohál et al., 2021). The accuracy and reliability of the results push the WGS toward a potential diagnostic application that is still under evaluation, but that could be pivotal in many clinical settings (Wang and Xing, 2023). A genome-driven approach provides a broader overview of the pathogen of interest, especially in case of uncommon and rare infections. In this scenario, bacterial isolates obtained from 20 positive blood cultures belonging to 20 individuals were investigated leading to the identification of an environmental NTM, the *Mycobacterium saskatchewanense* (Turenne et al., 2004). This study aims to highlight the importance of including *M. saskatchewanense* as a target in diagnostic assays and NTM surveillance in order to avoid misdiagnosing or underestimating the infection.

## 2 Materials and methods

### 2.1 Sampling

In 2023, 20 bacterial isolates of diagnostic unidentified NTM were isolated from positive blood cultures sequenced at the Operative Unit of Microbiology of the Greater Romagna Hub Laboratory, Italy. The isolates were recovered from each of the clinical anonymized leftover samples of 20 individuals. Initially, samples were processed during the daily diagnostic routine. Since no precise identification was obtained, the 20 isolates were processed using NGS.

### 2.2 Identification by hybridization

A first identification was performed by GenoType^®^ Mycobacterium CM (Hain Lifescience, Nehren, Germany) according to the manufacturer’s instructions. Although the manufacturer (Hain Lifescience, Nehren, Germany) indicates not to consider bands that appear faint or show a lower color intensity than the control bands, and given the possible cross-reactions of the MAC complex described for the GenoType^®^ Mycobacterium CM test, the GenoType^®^ Mycobacterium NTM-DR test (Hain Lifescience, Nehren, Germany) was performed as a second-level test.

### 2.3 MiSeq whole-genome next-generation sequencing and molecular identification

The DNA extraction was performed using the NucleoMag^®^ Pathogen Assay (Macherey-Nagel GmbH & Co. KG., Düren, Germany) designed for nucleic acid extraction and purification through paramagnetic beads. Samples were dosed on a Qubit™ fluorometer (Thermo Fisher Scientific Inc., Waltham, United States), and libraries were prepared using the Illumina DNA Prep kit (Illumina Inc., San Diego, United States). All the samples were sequenced on a MiSeq platform (Illumina Inc., San Diego, United States). Once having downloaded the FastQ files, the quality was checked using FastQC (Babraham Institute, Cambridge, United Kingdom). Since the dubious bands pattern from the first identification level, reads were trimmed and mapped on *Mycobacterium tuberculosis* reference genomes (NCBI accession number: NC-000962.3) using DNASTAR Lasergene software (DNASTAR Inc., Madison, United States) to confirm the identification in NTM. When identifying the species, the reference genome of *Mycobacterium saskatchewanense* was included in the analysis (Accession number: GCF_010729105.1) to detect mutations in *16S* and *hsp65* genes and calculate the Average Nucleotide Identity (ANI).

### 2.4 NextSeq whole-genome next-generation sequencing

Given the *M. saskatchewanense* rarity and to avoid error due to the sequencing method of choice, the 15 specimens (M02-23-03, M02-23-04, M03-23-03, M03-23-04, M04-23-01, M04-23-02, M04-23-03, M04-23-04, M05-23-01, M05-23-02, M05-23-03, M05-23-04, M06-23-01, M06-23-02, and M06-23-03) were also loaded on a NextSeq 2000 Illumina sequencer (Illumina Inc., San Diego, United States).

### 2.5 *De novo* genome assembly, genome annotation, and data acquisition

After trimming, reads were *de novo* assembled using SPAdes v.4.0.0 with the *–careful* option (Prjibelski et al., 2020). The quality of the assemblies was assessed both statistically, using QUAST v.5.2.0 (Mikheenko et al., 2023), and functionally, by evaluating genome completeness and the number of genes and their evolutionary relatedness to similar organisms, using BUSCO v.5.8.2 (Manni et al., 2021a). Only assemblies with a completeness of at least 95% were used for further analysis. To achieve chromosomal-level genome assemblies, scaffolds were reference-based assembled using RagTag v.2.1.0 (Alonge et al., 2022). Annotations of all assemblies were conducted with Prokka v.5.2.0 (Seemann, 2014). Once the *16S* and *hsp65* genes were identified in the isolates’ genomes, the Basic Local Alignment Search Tool (BLAST) performed the bacterial identification. The MLST was performed on locus L16, L19, L35, S12, S14Z, S19, S8, and S7 and the Sequence Type (ST) was defined on the PubMLST.org website (Jolley et al., 2018; Dinh-Hung et al., 2024). The MiSeq and NextSeq 2000 genome assemblies were checked with ResFinder v.4.6.0 for antibiotic-resistant mutations.

### 2.6 Cluster and phylogenetic analysis

Since no information is reported in the literature regarding the SNP limit to differentiate two clones of *M. saskatchewanense*, all samples sequenced on MiSeq and 10 samples (samples M02-23-01, M02-23-03, M02-23-04, M03-23-01, M03-23-02, M03-23-03, M03-23-04, M04-23-01, M04-23-02, and M05-23-04) sequenced on both MiSeq and NextSeq 2000 platforms were selected to define the error rate associated with the sequencing method of choice. Therefore, a cluster cgMLST analysis was performed on 5,711 genes using SeqSphere+ software (Ridom Bioinformatics GmbH, Germany).

To provide a comprehensive analysis, mutations across the complete genome were annotated in 15 samples sequenced on two Illumina platforms (samples M02-23-03, M02-23-04, M03-23-03, M03-23-04, M04-23-01, M04-23-02, M04-23-03, M04-23-04, M05-23-01, M05-23-02, M05-23-03, M05-23-04, M06-23-01, M06-23-02, and M06-23-03). Nucleotide mutation profiles were determined by aligning all sequences against the reference genome of *M. saskatchewanense* and subsequently annotated using Snipit v.1.6 (O’Toole et al., 2024).

The phylogenetic tree was constructed by concatenating the *16S*, *hsp65*, and *rpoB* genes and SNPs were annotated. Marker genes obtained from the genome assemblies were aligned using MAFFT v.7.525 (Katoh and Standley, 2013) with the *–auto* option. The resulting multiple-sequence alignment was examined and visualized with the Molecular Evolutionary Genetics Analysis (MEGA) software v.11.0.13 (Tamura et al., 2021). TrimAL v.1.5rev0 (Capella-Gutiérrez et al., 2009) was then applied to refine the alignment using the *gappyout* option. Finally, phylogenetic analysis was performed using IQ-TREE v.2.1.4 (Nguyen et al., 2015), employing the maximum likelihood method with ultrafast bootstrap set to 1,000 and automatic selection of the optimal nucleotide substitution model. The resulting tree was visualized with iTOL v.1.0 (Letunic and Bork, 2024). The rapid-growing *Mycobacterium abscessus* was selected as the outgroup for constructing the phylogenetic tree.

### 2.7 Principal component analysis

The principal component analysis (PCA) was performed on *16S, hps65*, and *rpoB* genes from sequenced samples and *Mycobacterium intracellulare, Mycobacterium lentiflavum, Mycobacterium simiae, Mycobacterium palustre, Mycobacterium heidelbergense, Mycobacterium avium, Mycobacterium scrofulaceum, Mycobacterium intermedium, M. abscessus*, and *M. saskatchewanense* that were divided into k-mer 5 nucleotides long using FastK v.1.1.0 (Myers, 2025) freely available on GitHub. PCA was performed using Stata Statistical Software: Release 18 (STATA, College Station, TX, StataCorp LLC).

### 2.8 Statistics

The total number of contigs, the total length, and N50, the total number of contigs, the total assembled length, the average coverage, the percentage of C-G bases, and the number of undefined bases obtained from the analysis of MiSeq and NextSeq 2000 were compared to determine the best performance for investigating unknown bacterial agents. The results of the cluster analysis were organized in a hierarchical cluster analysis dendrogram using STATA.

## 3 Results

### 3.1 Identification by hybridization

**Figure 1:**
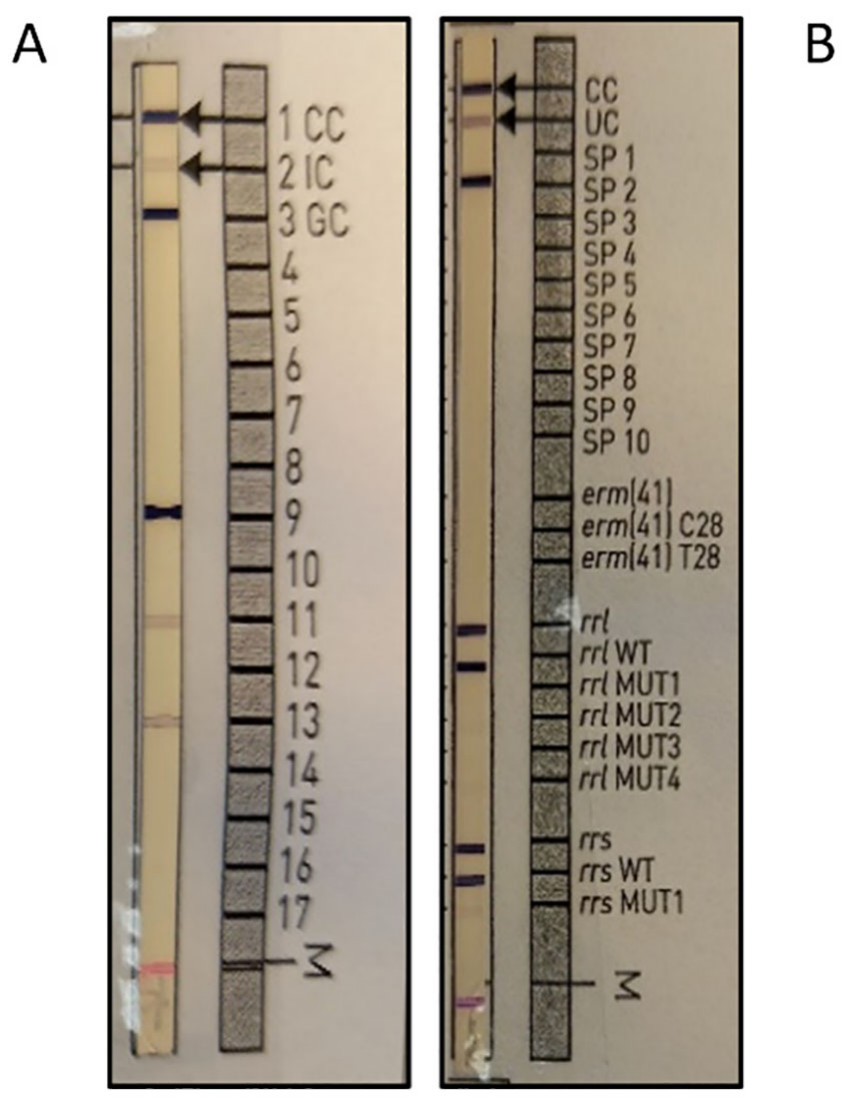
Hybridization pattern obtained for the 20 isolates of *Mycobacterium saskatchewanense*. **(A)** Results obtained from the GenoType^®^ Mycobacterium CM assay where band 9 was typic of *Mycobacterium intracellulare* and bands 11 and 13 are weakly colored. **(B)** Results obtained from GenoType^®^ Mycobacterium NTM-DR confirming the identification of *Mycobacterium intracellulare*.

The identification performed with the GenoType^®^ Mycobacterium CM assay revealed a peculiar banding pattern: band 9, typically associated with *Mycobacterium intracellulare*, was clearly present; bands 11 and 13 were weakly visible and did not match with any other species included in the assay (**Figure 1**). Similarly, the results of GenoType^®^ Mycobacterium NTM-DR confirmed the identification of *M. intracellulare* species.

### 3.2 Identification by 16S and hsp65

The *16S* sequence was identified in the assembled genomes and confirmed with BLAST: The examination resulted in 100% homology between the reference *M. saskatchewanense 16S* RNA sequence and the query genomes. The second-level identification inferred from the *hsp65* gene confirmed the previous one.

### 3.3 Whole genome

From the MLST analysis, the locus pattern (L16 369; L19 331; L35 310; S12 318; S14Z 279; S19 278; S8 352; S7 missing) and ST 691 was the same for all the samples. Additionally, no resistant mutations were found in genome assemblies from MiSeq and NextSeq 2000 Illumina platforms. According to the BUSCO analysis, the overall gene score was 99.3% referring to the *M. saskatchewanense* species for isolates sequenced on the NextSeq 2000 platform, whereas 99.2% was obtained for sequences obtained from MiSeq.

Due to the similarity in the MLST results, the level was deepened to a whole-genome investigation by an ANI approach between the GCF_010729105.1 and genome assemblies. ANI results showed that the samples’ diversity from the reference genome ranged from 0.04% to 0.035%, whereas the differences among specimens varied from 0.001% to 0.006%. In the end, the identity between the reference sequence and the query genomes was approximately 96%; on the other hand, the sample sequence identity was approximately 99.9%. Interestingly, sample M03-23–03 had a lower percentage of identity when compared with the different samples (99.5%) (**Supplementary Figure S1**).

**Figure 2:**
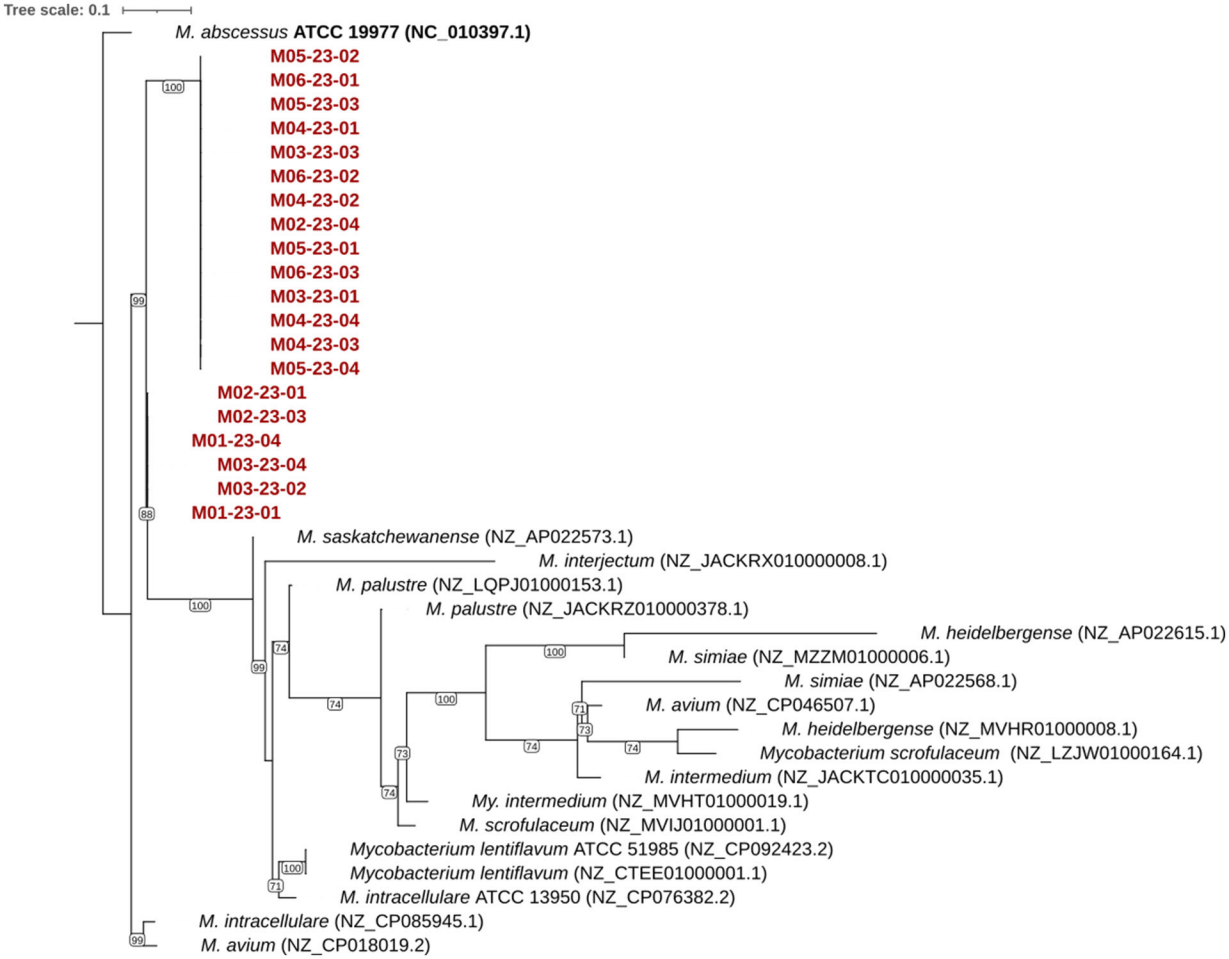
The Maximum-Likelihood phylogenetic tree. The tree was constructed based on the concatenated 16S, hsp65, and rpoB from slow-growing *Mycobacterium* species, including isolate 20 *Mycobacterium saskatchewanense* (highlighted in red). The tree was rooted using the rapid-growing *Mycobacterium abscessus* ATCC 19977 as outgroup. Only bootstrap values greater than 70 are shown. The tree was inferred using IQ-TREE v.2.1.4, employing automated model selection (TVM+F+R4) and 1,000 bootstrap replicates.

According to the phylogenetic analysis, the 20 isolate sequences on the MiSeq platform clustered as related species (**Figure 2**).

Since no phylogenetic distance is reported in the literature to distinguish different clones for *M. saskatchewanense*, a cluster analysis graph was generated for the 20 samples sequenced on the MiSeq Illumina platform using the cgMLST. A total of 592 alleles were excluded from the study because they were not represented in the genome assemblies. The cgMLST showed that samples having one different allele were M06-23-03/M06-23-02, M02-23-03/M03-23-04, M05-23-03/M06-3-01, and M05-23-01/M01-23-04. Conversely, samples M02-23–01 and M03-23–03 resulted in an average of 18.5 and 25.6 allele differences, respectively, when compared with other isolates, making them the most divergent within the dataset. Considering the whole tested population, the average number of different alleles between isolates is 12.3 (**Supplementary Figure S2**).

### 3.4 MiSeq and NextSeq chemistry comparison

**Figure 3:**
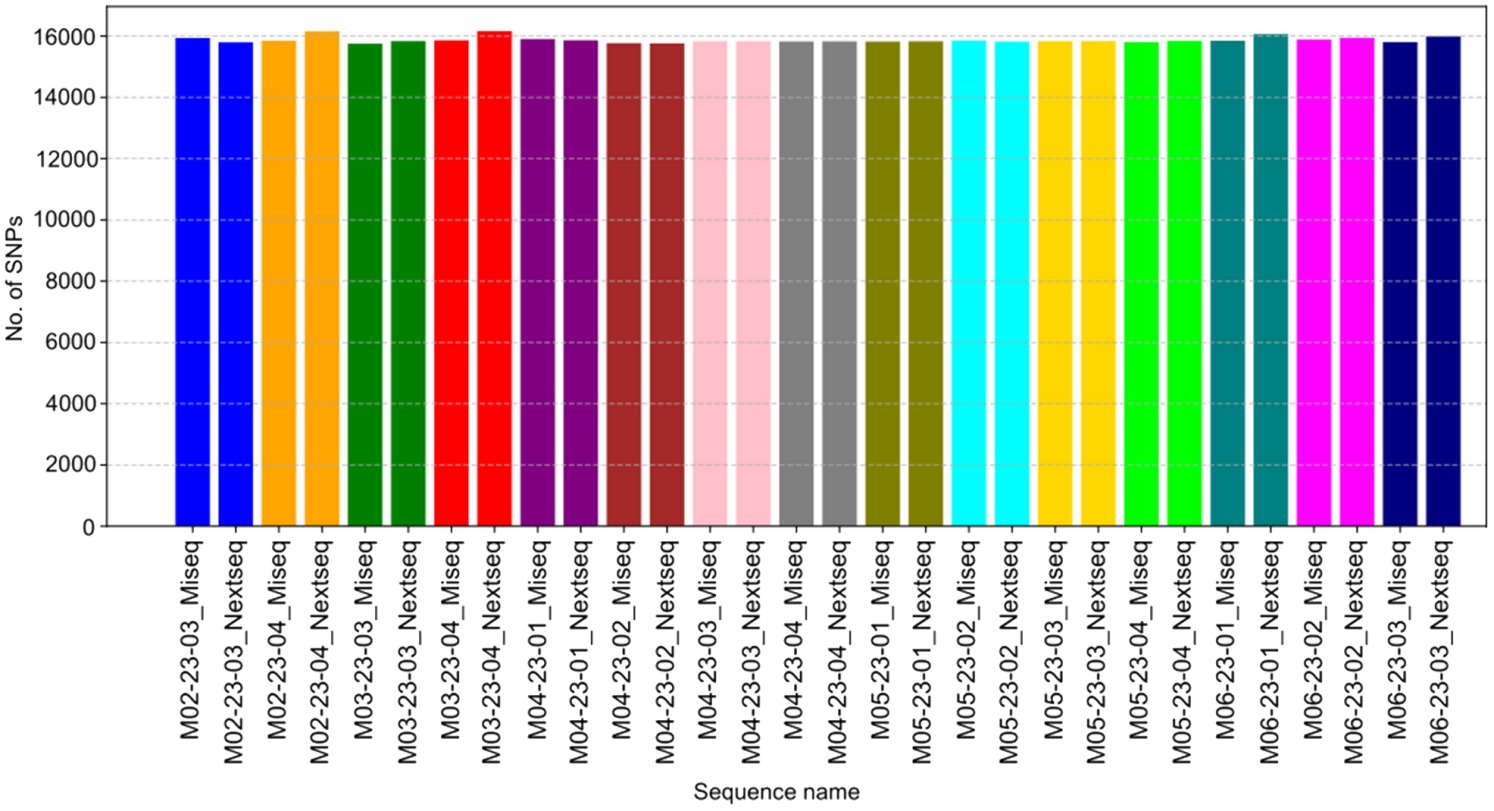
Bar plot of the number of single-nucleotide polymorphisms (No. of SNPs). The SNPs were counted in 15 samples sequenced on MiSeq and NextSeq 2000 Illumina platforms.

To evaluate the impact of the two Illumina platforms implied in the study, the assembly metrics of the 15 samples were analyzed to define which platform could be more feasible to sequence uncommon mycobacteria. The parameters considered were N50, the total number of contigs, the total assembled length, the average coverage, the percentage of C-G bases, and the number of undefined bases (**Supplementary Table S1**). Given the different sequencing chemistry of MiSeq and NextSeq 2000, the number of mutations in the complete genome was compared, resulting in around 160,000 SNPs for both platforms. Although NextSeq 2000 annotated a higher number of SNPs, in three cases (samples M02-23-03, M04-23-01, and M05-23-02), the MiSeq assemblies contained more mutations For samples M04-23-02, M04-23-03, M04-23-04, M05-23-01, and M05-23-03, the percentages of reported SNPs were comparable between platforms (**Figure 3**).

When examining SNP count differences, NextSeq 2000 consistently annotated more mutations overall, with an average discrepancy of approximately 95 SNPs in the whole genome. The unique mutations for each sample were also considered. The majority relied on AG, CG, CT, GA, and GC SNPs, whereas AT and TA were less frequent. Non-shared SNPs were distributed equally in the genome of samples M02-23-01, M03-23-01, M03-23-04, M04-23-01, M04-23-02, M05-23-04, M06-23-01, and M06-23-03, whereas mutations located at the ending positions—from 10^5^ to 10^6^ genome locations—in samples M02-23-03, M04-23-03, M04-23-04, M05-23-01, M05-23-02, M05-23-03, and M06-23-02. Notably, the mutations reported in MiSeq assemblies were located mainly in the ending genome positions (**Supplementary Figure S3**). A cgMLST complete concordance was obtained for the sample M05-23-04. Samples M04-23–02 and M03-23–01 were found to have one different allele if sequenced on different platforms, whereas M02-23–04 sequenced on the MiSeq platform resulted in having two different alleles from its correspondence on NextSeq 2000. Interestingly, sample M02-23–03 differed from M03-23–04 sequenced on MiSeq by one allele, two alleles from M03-23–04 sequenced on NextSeq 2000, and three alleles from M02-23–03 sequenced on NextSeq 2000. In the same manner, sample M03-23–04 has one allele different from M02-23–03 sequenced on MiSeq, two different alleles from its NextSeq 2000 correspondence, and four different alleles from M03-23–01 and M04-23–02 sequenced on MiSeq (**Supplementary Figure S4**).

From the cgMLST, the mean rate of detected different alleles in each sample was calculated for both sequencers. Each sample was compared pairwise, resulting in 45 total comparisons (10 samples per nine comparisons each). The mean allele difference for each sample detected from MiSeq is 15.55, whereas 15.73 was calculated for NextSeq 2000. Among the 45 comparisons, NextSeq 2000 identified more allele differences in 25 cases, whereas MiSeq reported more differences in 16 cases. Complete concordance was observed in four comparisons (samples M04-23-01/M02-23-03, M02-23-01/M03-23-02, M02-3-01/M03-23-03, and M03-23-04/M03-23-03). Therefore, considering the difference between the sequencers, the error rate associated with the technique is +/− 1.8 alleles. Regarding phylogenetic diversity, using two different sequencing techniques has been observed not to impact phylogenetic diversity (**Supplementary Figures S5**).

### 3.5 Principal component analysis and SNP annotation

**Figure 4:**
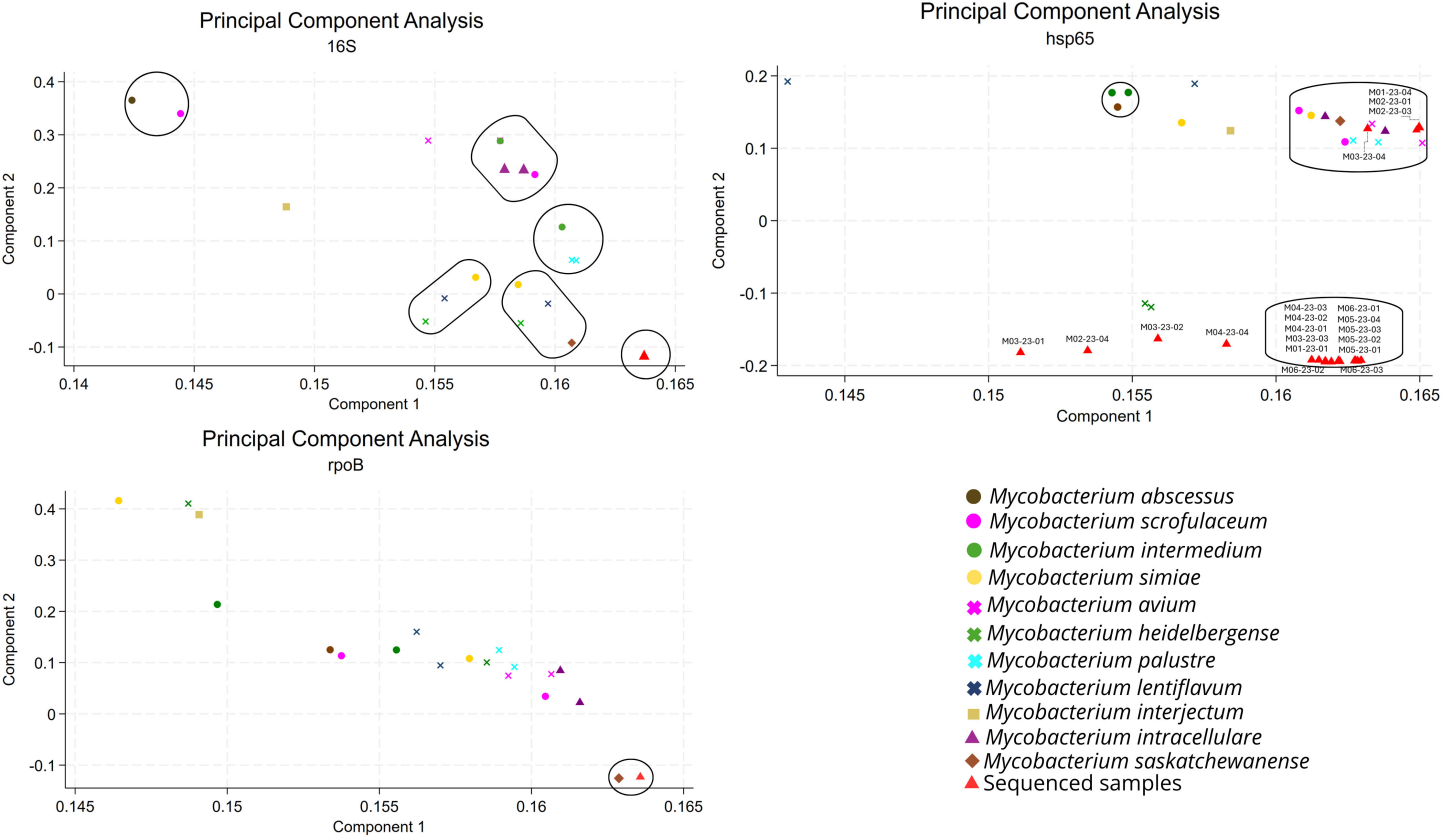
Principal component analysis (PCA) of the 16S, hsp65, and rpoB genes. The analysis included the 20 *Mycobacterium saskatchewanense* samples sequenced on the MiSeq Illumina platform, the *M. saskatchewanense* reference genome, and 10 nontuberculous mycobacteria species.

The PCA performed on *16S, hsp65*, and *rpoB* highlighted that the latter gene can be used to differentiate *M. saskatchewanense* from other NTM species. Although both *16S* and *rpoB* obtained from the 20 samples clustered together and closely to the *M. saskatchewanense* reference genome, *rpoB* showed a tighter association and a clear separation from other NTM sequences (**Figure 4**).

**Figure 5:**
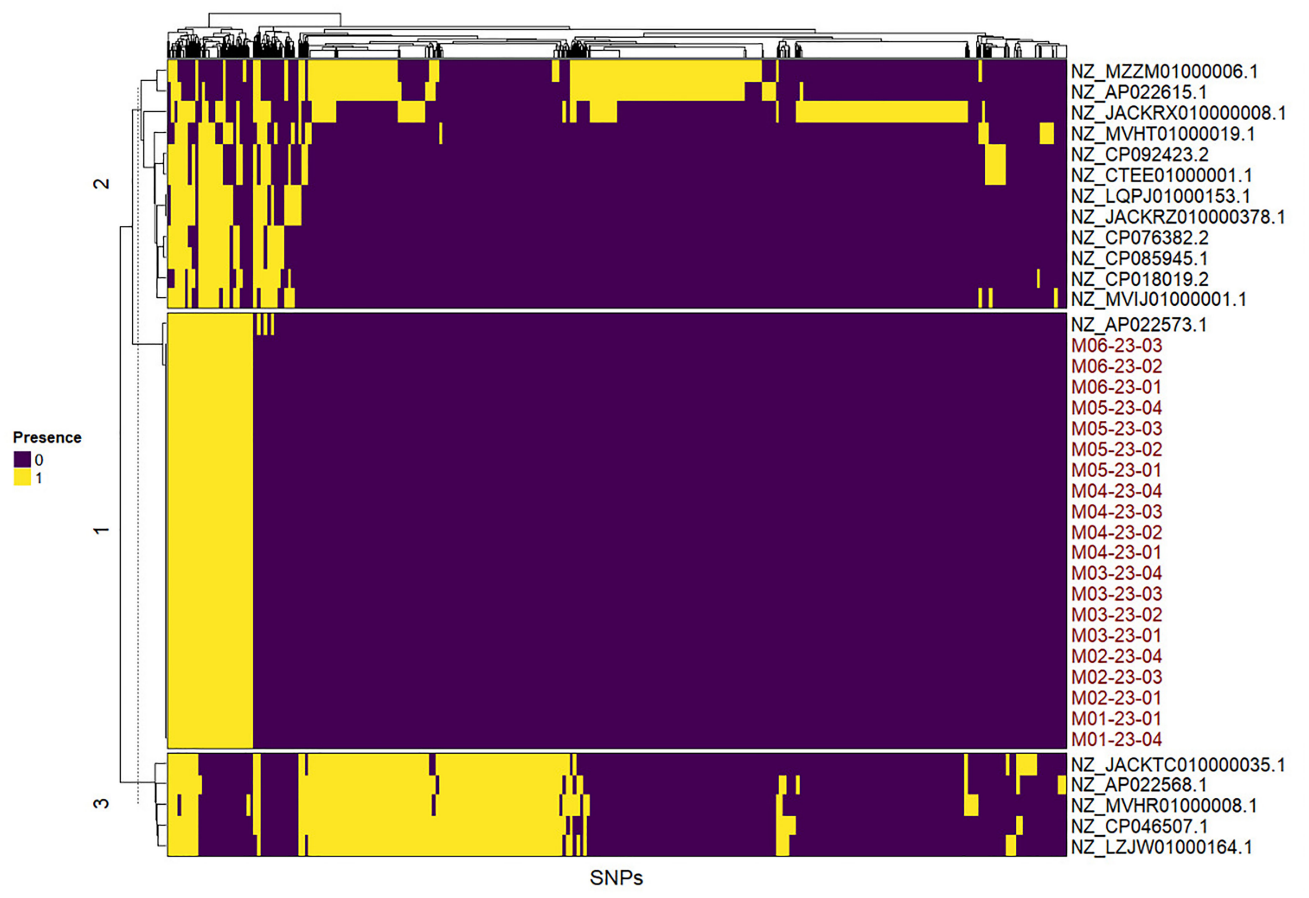
Heatmap obtained from the single nucleotide polymorphisms analysis of rpoB gene. The mutations were classified as present (1) or absent (0) in 20 *Mycobacterium saskatchewanense* samples, the *M. saskatchewanense* reference genome, and 10 non-tuberculous mycobacteria species.

SNP analysis of the three genes confirmed that *rpoB* offers a more distinct clustering of *M. saskatchewanense* compared with 16S and *hsp65* (**Figure 5**; **Supplementary Figure S6**).

## 4 Discussion

Accurate identification of infectious agents is crucial for diagnosis, epidemiological investigations, and clinical intervention The presence of multiple species belonging to the same genus or complex can further complicate diagnostic interpretation and hinder the reconstruction of infection patterns (Moghaddam et al., 2022). In the field of mycobacteria research, NTMs were long considered environmental contaminants and remain largely neglected (Gcebe and Hlokwe, 2017). More recently, NTMs have increasingly been reported as infectious agents in immunocompromised patients, and novel *Mycobacterium* strains have been discovered (Iversen et al., 2025). Traditional microbial culture showed several limitations; therefore, the introduction of PCR-based techniques, NGS, and algorithms of analysis expanded the knowledge of uncommon NTMs, overcoming the limit for clinical and laboratory applications (Ghodousi et al., 2023; Huang et al., 2023). Indeed, technical challenges in NTM identification are well-reported in the literature and often delay the diagnosis and increase the costs due to prolonged hospitalizations and treatment (Lee et al., 2023). Sequencing of conserved regions such as *16S* and *hsp65* genes has proven valuable in supporting the interpretation of diagnostic tests, particularly in ambiguous cases (Tortoli, 2014; Solaghani et al., 2024). More recently, NGS and WGS have improved laboratory and diagnostic capabilities, enabling the analysis of hundreds of NTM genomes and providing a comprehensive approach for investigating genetic diversity and antibiotic resistance. The application of WGS in clinical settings may therefore help overcome current diagnostic and culture-associated limitations (He et al., 2020). Given the previously uncertain diagnostic results, the authors sought to investigate the genomic diversity of 20 *M. saskatchewanense* isolates to expand the scientific knowledge about this rare and recent NTM species (Turenne et al., 2004). To achieve this, an MLST approach was used as a first-level method to characterize *M. saskatchewanense* and investigate evolution patterns (Wuzinski et al., 2019). All isolates were classified in ST 691. To the authors’ knowledge, no specific MLST pattern or ST had been previously reported for *M. saskatchewanense*. Interestingly, allele S7 was absent in all the isolates, suggesting the need for further studies on the genomic organization of *M. saskatchewanense* to define possible alternative locus sequences. This represents the first report describing the MLST pattern and ST of *M. saskatchewanense*. Nevertheless, the analysis of multiple genes through WGS remains the preferred approach for resolving the phylogeny of unknown or poorly studied NTM (Fedrizzi et al., 2017). For environmental NTMs, a precise identification and characterization of closely related species is not always possible and genomic studies often lack a standardized methodology. Therefore, the investigation on genetic diversity and the impact of the NGS technology are still an open debate (Fedrizzi et al., 2017; Mugetti et al., 2021). To distinguish clonal populations, the cgMLST was applied in mycobacteria studies, completing the MLST approach and achieving highly consistent results in phylogenetic analysis (Menghwar et al., 2022). In our study, the cgMLST cluster analysis identified approximately 97.8% of *M. saskatchewanense-*specific genes across the 20 genome assemblies (Manni et al., 2021b). This result also correlated with the percentage of whole genome identity between the isolates and the reference sequence. Unlike the 98% identity that was used for other NTM (Behra et al., 2022), *M. saskatechewanese* isolates can be classified as the same species also when the percentage of identity results in nearly 96% (Turenne, 2019). The concatenated analysis of *16S*, *hsp65*, and *rpoB* genes performed on MiSeq and NextSeq 2000 genome assemblies revealed no differences in phylogenetic tree arrangement, confirming the robustness of the method. The application of PCA to mass spectrometry spectra has already been used in mycobacteria identification, proving to be a valid method to differentiate between species and improve accuracy in classification (Kehrmann et al., 2016; Hanson et al., 2017). Following this rationale, in our study, PCA was applied to genome sequences to further investigate the clustering patterns observed through concatenated genes phylogenetics. PCA based on *16S* and *rpoB* genes successfully clustered different NTM species, whereas *hsp65* resulted in a more scattered distribution. Interestingly, the PCA results were supported by the SNP analysis which confirmed *rpoB* as the more suitable gene for distinguishing *M. saskatchewanense*. This suggests that, in case of ambiguous diagnostic results for *M. intracellulare*, a molecular confirmation test targeting *rpoB* can be implemented. The sequence diversity between the reference genome and the isolates resulted in approximately 3%, consistent with the literature (Turenne, 2019). The cgMLST results aligned to the phylogenetic data, suggesting high concordance between the two approaches. Since the molecular surveillance of *M. tuberculosis* is commonly performed using cgMLST to ensure inter-laboratory comparability (Diricks et al., 2022), our findings confirm that the same molecular method can be applied to *M. saskatchewanense*. Assessing the number and distribution of SNPs across the whole genome is necessary for proper genomic characterization. This study provides a proof of concept on how a sequencer’s chemistry can affect the final molecular epidemiological results. NextSeq 2000 generally reveals mutations along the whole genome, whereas MiSeq concentrates SNPs at the end of the sequence. NextSeq 2000 also recorded a higher overall SNP count, with an estimated average technical variation of approximately 1.8 alleles between platforms. The higher efficiency of NextSeq 2000 was confirmed by comparing the assembly outputs with the ones obtained from MiSeq (Browne et al., 2020). Despite the similar C-G content, the NextSeq 2000 assemblies presented fewer contigs and increased N50 (Gurevich et al., 2013). The SNPs’ analysis of the 15 samples sequenced with both platforms corroborated the previous data: NextSeq 2000 revealed approximately 95 SNPs per sample more than MiSeq across the whole genome. Focusing on the unique SNPs, mutations were equally distributed across the genome using the NextSeq 2000 approach. This study is relevant to the Italian context, where the environmental and clinical manifestations of *M. saskatchewanense* have been reported in recent years. In 2019, the first Italian infection of *M. saskatchewanense* was documented in a solid-organ transplant patient with chronic renal disease (Mento et al., 2019). Subsequently, *M. saskatchewanense* was identified at the environmental level in 17 dialysis fluids from the Emilia-Romagna region (Bisognin et al., 2023). This draws attention to the lack of proper surveillance for environmental contamination. A second Italian study screened 722 ultrapure dialysis fluid samples in the same region and detected 35 positive cases. The isolates were then sequenced and analyzed, highlighting the importance of dedicated environmental screening and disinfection (Cannas et al., 2024). Our findings are consistent with these reports and strongly support the inclusion of *M. saskatchewanense* in diagnostic and surveillance panels. Moreover, the growing concern of NTM and *M. saskatchewanense* understanding was also confirmed by the introduction of the pathogen in one genomic comparison analysis for NTM identification (van Ingen et al., 2009). The identification of proper methodology and threshold proposed in this analysis may improve the diagnostic interpretation. The application of WGS in genomic epidemiology remains under discussion. Collaborative efforts between laboratories are recommended to integrate genomic data for European surveillance and preparedness strategies (Struelens and Brisse, 2013). Conor et al. highlighted the need to validate and standardize WGS protocols, bioinformatic pipelines, and phylogenetic analysis for *M. tuberculosis* investigation in both clinical and public health settings (Meehan et al., 2019). Similar standardization is essential also for NTM. Our study can contribute to this effort to define technical limitations associated with current sequencing chemistries. Finally, given the limited availability of *M. saskatchewanense* complete genomes in public databases, our study contributes by depositing raw data of 20 isolates that can be reassembled and compared with further investigations. Continued pathogen discovery and genome characterization expand the knowledge and improve molecular technique applications. To date, scientific literature on NTMs remains limited; therefore, this study attempted to introduce a new perspective on the diagnostics and genomic analysis of the *M. saskatchewanense*. Library preparation protocols and bioinformatic pipelines should be implemented and standardized to advance NTM research. Genomic analysis provides a foundation for the development of diagnostic assays aimed at preventing misdiagnosis and strengthening the surveillance of rare and atypical NTM species.

## Data Availability

The datasets presented in this study can be found in online repositories. The names of the repository/repositories and accession number(s) can be found below: https://www.ncbi.nlm.nih.gov/, PRJNA970246.

## Supplementary material

Supplementary Table 1 assembly parameters of the samples sequenced on MiSeq and NextSeq 2000 Illumina platform. The parameters considered in the analysis are the number of contigs, N50, total assembled length, the average coverage, the percentage of C-G bases, and the number of undefined bases. #: number.
