# Supplementary material for: NGS-based approach for diagnostically unidentified Mycobacterium saskatchewanense, a rare non-tuberculous mycobacterium
Source: Front Cell Infect Microbiol. 2025 Dec 16;15:1685898. doi: 10.3389/fcimb.2025.1685898 (PMC12748190; doi:10.3389/fcimb.2025.1685898)
Supplement: Supplementary file 2 [file DataSheet1.docx]

Supplementary Material

# Supplementary Data

Supplementary material of the *NGS-based approach for diagnostically unidentified Mycobacterium saskatchewanense, a rare Nontuberculous mycobacterium* manuscript. Images are listed in the present word file. The Supplementary table (Table S1) is submitted as Excel file because it exceeds the page dimension.

# Supplementary Figures

## Supplementary Figure 1


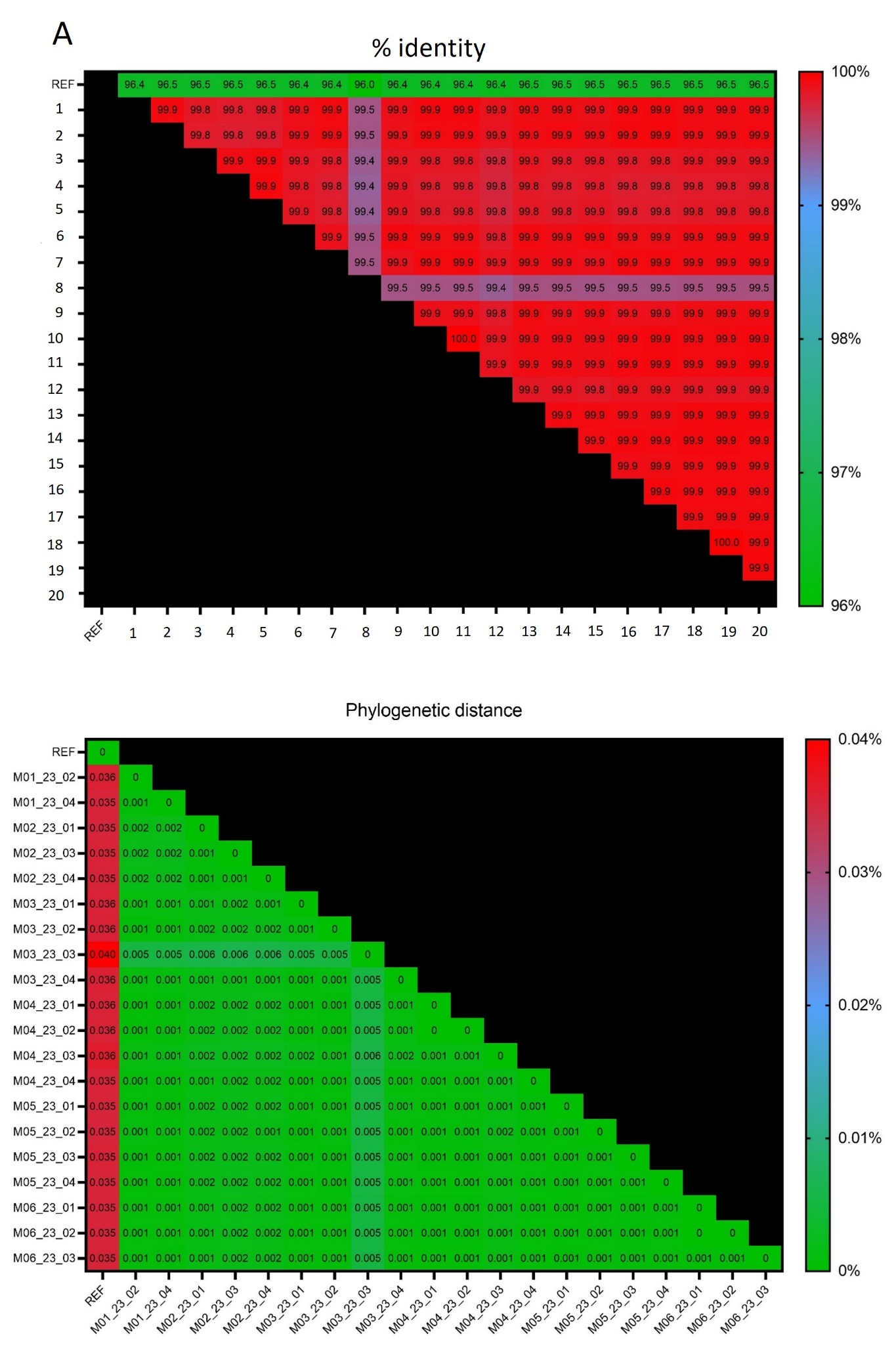


**Supplementary Figure 1.** The matrix reporting the percentage of identity and diversity. A: Percentage of Average Nucleotide Identity between the 20 *Mycobacterium saskatchewanense* samples sequenced on the MiSeq Illumina platform; B: Percentage of diversity between the 20 *Mycobacterium saskatchewanense* samples sequenced on the MiSeq Illumina platform. REF = *Mycobacterium saskatchewanense* reference genome (Accession number: NZ-AP022573.1).

**2.2 Supplementary Figure 2**


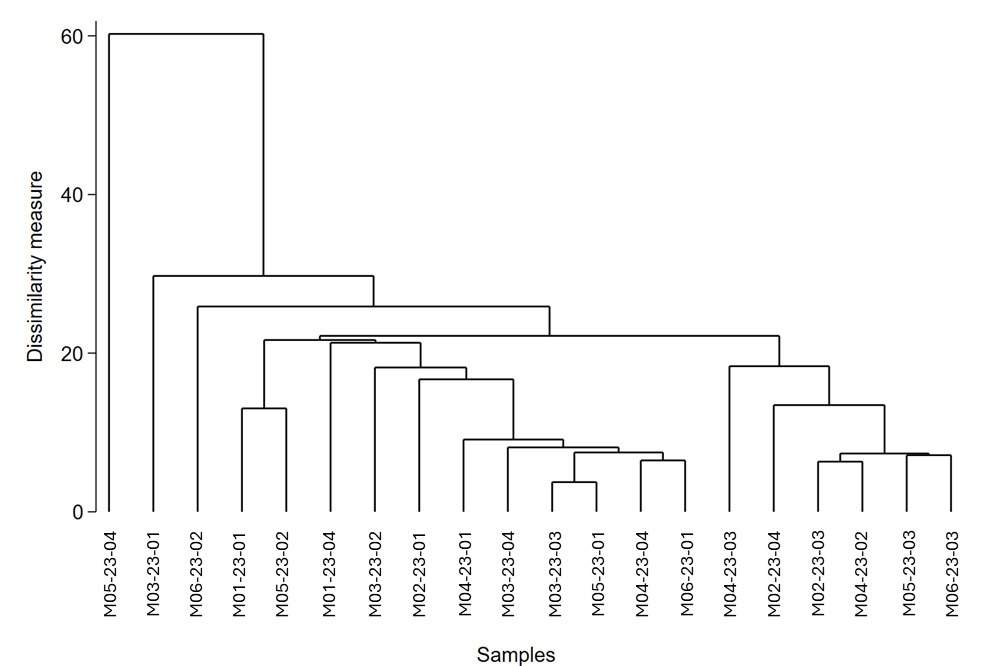


**Supplementary Figure 2**. Hierarchical cluster analysis. 20 samples of *Mycobacterium saskatchewanense* sequenced on the MiSeq Illumina platform. M: samples sequenced through the MiSeq platform. N: samples sequenced through the NextSeq 2000 platform.

## 2.3 Supplementary Figure 3


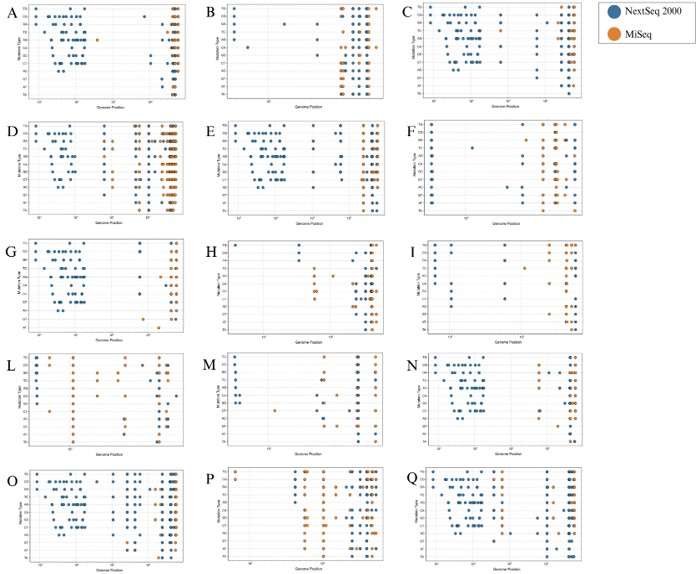


**Supplementary Figure 3.** Unique mutation annotated in 15 samples sequenced on MiSeq and NextSeq 2000 Illumina platforms. The Single Nucleotide Polymorphisms were classified according to their position in the genome and the base change. A: M02-23-01; B: M02-23-04; C: M03-23-03; D: M03-23-04; E: M04-23-01; F: M04-23-02; G: M04-23-03; H: M04-23-04; I: M05-23-01; L: M05-23-02; M: M05-23-03; N: M05-23-04; O: M06-23-01; P: M06-23-02; Q: M06-23-03.

## 2.4 Supplementary Figure 4


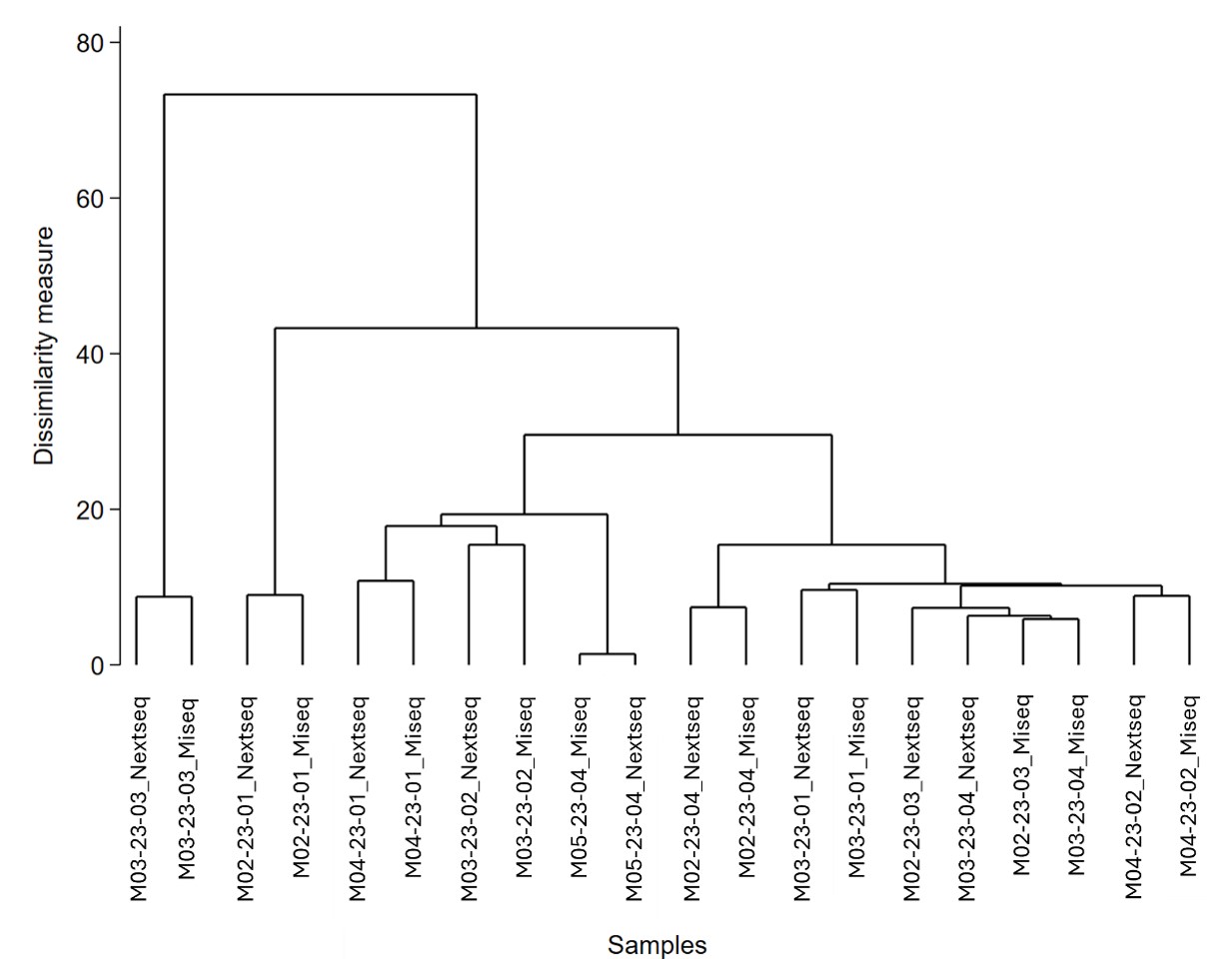


**Supplementary Figure 4.** Hierarchical cluster analysis. 10 samples of *Mycobacterium saskatchewanense* sequenced on MiSeq and NextSeq 2000 Illumina platforms. M: samples sequenced through the MiSeq platform. N: samples sequenced through the NextSeq 2000 platform.

## 2.5 Supplementary Figure 5


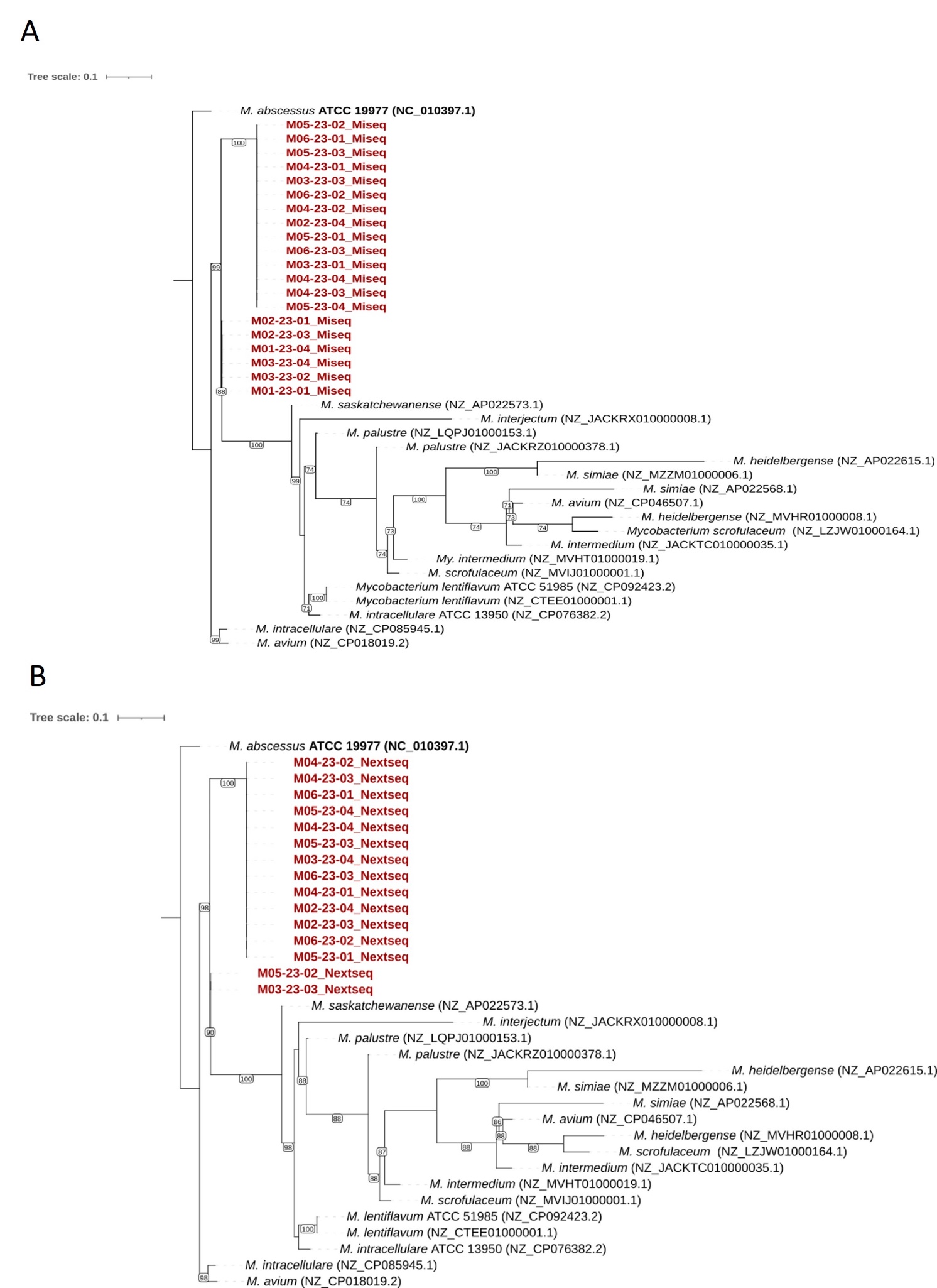


**Supplementary Figure 5.** Phylogenetic analysis of *16S, hsp65*, and *rpob* concatenated genes of the 15 *Mycobacterium saskatchewanense* isolates (highlighted in red) sequenced. The output was defined as *Mycobacterium absessus* ATCC 19977 and additional Mycobacteria species were added (accession number in parentheses). The tree was displayed with IQ-tree v.2.3.6. A: samples sequenced on MiSeq Illumina platform. B: samples sequenced on NextSeq 2000 Illumina platform.

## 2.6 Supplementary Figure 6


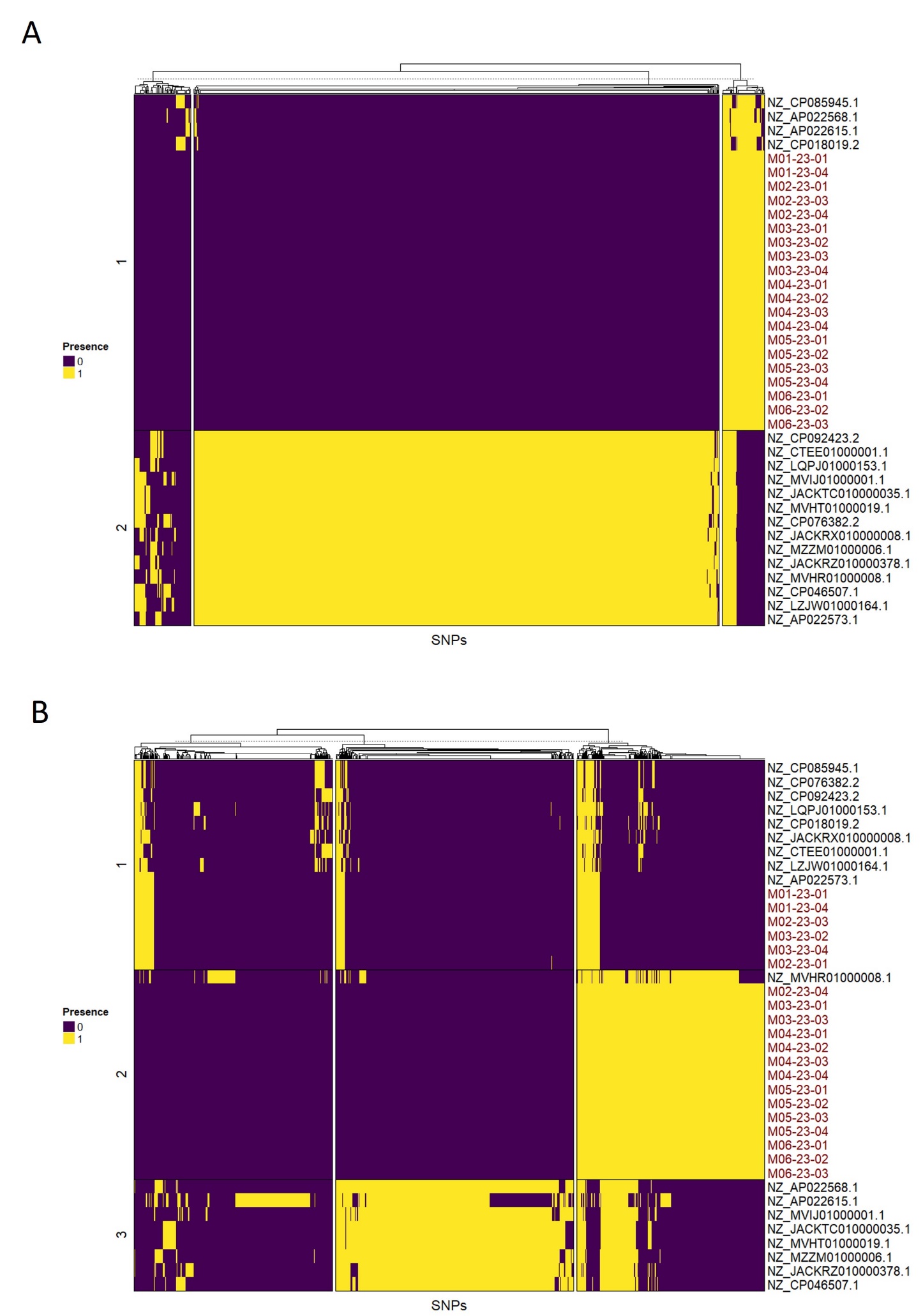


**Supplementary Figure 6.** Heatmap obtained from the single nucleotide polymorphisms analysis of *16S* and *hsp65* genes. The mutations were classified as present (1) or absent (0) In 20 *Mycobacterium saskatchewanense* samples, the *M. saskatchewanense* reference genome, and 10 Nontuberculous mycobacteria species. A: 16S gene; B: hsp65 gene
